# Pediatric Diastolic Heart Failure: Clinical Features Description of 421 Cases

**DOI:** 10.3389/fped.2022.846408

**Published:** 2022-05-02

**Authors:** Bo Pan, Di Hu, Huichao Sun, Tiewei Lv, Wangguo Xu, Jie Tian

**Affiliations:** ^1^Department of Cardiology, Children’s Hospital of Chongqing Medical University, National Clinical Research Center for Child Health and Disorders, Ministry of Education Key Laboratory of Child Development and Disorders, Chongqing, China; ^2^Chongqing Key Laboratory of Pediatrics, Chongqing, China; ^3^Department of Otorhinolaryngology, Children’s Hospital of Chongqing Medical University, Chongqing, China; ^4^Department of Cardiology, Yongchuan Hospital of Chongqing Medical University, Chongqing, China

**Keywords:** pediatric, heart failure, diastolic heart failure, BNP, systolic heart failure

## Abstract

**Background and Aim:**

Heart failure in children differs substantially from the adult population. Clinical characteristics of pediatric diastolic heart failure has rarely been reported. In this study, we aimed to summary the causes, clinical features, lab tests, and treatment effect of pediatric diastolic heart failure.

**Methods:**

This study was a single center, retrospective study conducted in Children’s Hospital of Chongqing Medical University. Children who were diagnosed with diastolic heart failure (DHF) without systolic heart failure (SHF) between 2006 and 2014 were included. Meanwhile, SHF (without DHF) cases were also collected from 2013 to 2014.

**Results:**

A total of 421 DHF and 42 SHF cases were included. The average age of pediatric DHF was 1.89 ± 3.29 years old, significant younger than that of SHF (4.65 ± 4.90). The top three cardiovascular causes of DHF were complex congenital heart malformations (53.4%), simple congenital heart defect (15.7%), and cardiomyopathy (7.4%). Alternatively, number of cardiomyopathy cases (57.1%) ranked first in SHF group. Simple congenital heart diseases (CHDs) rarely caused SHF. The most common symptom and sign were tachypnea and hepatomegaly in pediatric HF. Symptoms like cyanosis, feeding difficulty, be fidgety, pale, fatigue, and edema were valuable in differential diagnosis of DHF and SHF in children. B-type natriuretic peptide (BNP) increase was found in 36.9% of DHF children, and 60% in SHF patients. Sensitivity of BNP greater than 100 pg/ml in diagnosis of DHF was 0.37, and specificity of it was 0.86. Diastolic function indicators, such as E/A (early wave/late wave) ratio, IVRT (isovolumic relaxation time) were significant recovered after treatment in DHF patients. Less therapeutic benefits were achieved in children with cardiomyopathy induced DHF, in compared with non-cardiomyopathy patients.

**Conclusion:**

Pediatric DHF and SHF were largely different in primary causes, clinical symptoms and signs and short-term prognosis. There was a limit diagnostic value of BNP with 100 pg/ml as cut-off value in pediatric DHF. Larger, multicenter studies of pediatric DHF are required in the future.

## Introduction

Heart failure in children is a serious public health concern, as the costs are considerably higher for children than adults because of the frequent need for surgical or catheter-based intervention (1). The demands of medical care can fray the family structure and adversely affect parental economic productivity (1). However, heart failure in children has been poorly studied and understood. It is widely acknowledged that pediatric heart failure is largely different from that in adults (2), both in clinical characteristics and pathological mechanisms, for at least two reasons. Firstly, the basic diseases of pediatric heart failure are considerably different from adults’ (3); secondly, mammalian hearts undergo maturation period after birth (4). At present, the accurate time point of human cardiomyocytes maturation is still not clear. Some studies considered cardiac maturity occurs in puberty (5). Therefore, data and conclusions from adult heart failure cannot be simply applied in pediatric patients. There is a strong need of studies of children heart failure.

Diastolic heart failure has been referred to as heart failure with preserved ejection fraction (HFpEF) in recent years, and is characterized by signs and symptoms of heart failure and a left ventricular ejection fraction (LVEF) greater than 50% (6). Diastolic heart failure (DHF) is more common among females and elderly population. Abnormalities of DHF both in active ventricular relaxation and passive ventricular compliance, could lead to a decrease in stroke volume and cardiac output (7). In adult patients, DHF is mainly caused by hypertension, diabetes, and coronary heart diseases (8). Measurement of B-type natriuretic peptide (BNP) is considered as a useful biomarker in diagnosis of DHF, and valuable in severity evaluation (9). Multiple trials have not found medications to be an effective treatment, even medications that are effective in patients with heart failure and a reduced ejection fraction have generally not been useful in patients with HFpEF (10). However, DHF in children has rarely been studied. In this retrospective study, clinical features, and treatment effect of 421 DHF and 42 systolic heart failure (SHF) cases were summarized and compared. Data from this study will provide more information on pediatric DHF and might contribute to the understanding of pediatric DHF for pediatricians.

## Materials and Methods

This was a retrospective study conducted in Children’s Hospital of Chongqing Medical University, National Clinical Research Center for Child Health and Disorders. This study was approved by the Institutional Review Board of Children’s Hospital of Chongqing Medical University, Chongqing, China.

A total of 421 DHF cases from October, 2006 to December, 2014 were collected. Meanwhile, 42 SHF cases from January, 2013 to December, 2014 were included. All those clinical data: general information, basic diseases, symptoms, signs, echocardiography reports, BNP levels, and treatments were summarized. Plasma BNP levels of 979 patients (all hospitalized patients in department of pediatric cardiology) form January, 2013 to December, 2014 were collected. BNP detection in our center was started in 2010, so number of DHF patient with BNP result was 130.

Inclusion criteria:

Diastolic heart failure: (1) to have heart failure symptoms and signs, Modified Ross Score (11) greater than or equal to 3 points; (2) LVEF greater than 50%;

Systolic heart failure: (1) to have heart failure symptoms and signs, Modified Ross Score greater than or equal to 3 points; (2) LVEF less than 50%.

### Statistical Analysis

The categorical data and continuous data were analyzed by χ^2^, Fisher’s Exact test and *t*-test, respectively. Measurement data expressed as mean ± standard deviation (x ± s). Statistical analysis was performed using SPSS (version 23.0, IBM Corporation, Armonk, NY, United States). *p*-Value less than 0.05 was considered statistically significant.

## Results

### General Information of Diastolic Heart Failure and Systolic Heart Failure Patients

As shown in Table 1, there were 421 DHF patients in total, the number of boys (250) was significant more than that of girls (171). On the contrary, females were slightly more common in SHF patients (no significant differences). The average age of DHF patients was 1.81 ± 3.29 years, significantly lower than that of SHF patients (4.65 ± 4.90), *p* < 0.001.

**TABLE 1:** General information of pediatric DHF and SHF patients.

General information	DHF group	SHF group	χ^2^	*p*-Value
Total numbers	421	42	/	/
Age (years)	1.89 ± 3.29	4.65 ± 4.90	5.06	0.000
M/F	250/171	16/26	7.08	0.008

*Values are expressed as mean ± SE for each group. DHF, diastolic heart failure; SHF, systolic heart failure; M, male; F, female.*

### Causes of Diastolic Heart Failure and Systolic Heart Failure in Children

Primary diseases of pediatric DHF and SHF were illustrated in Table 2. Congenital heart disease (CHD) ranked first in all DHF primary diseases, nearly 70%, followed by cardiomyopathy (7.4%) and arrhythmia (5.9%). Notably, most CHD cases were complex ones, here “complex” was defined as two or more cardiovascular malformations in one patient. Details about the spectrum of complex CHD was shown in Supplementary Table 1. In simple CHDs, ventricular septal defect was the most common causes of DHF, even slightly higher than cardiomyopathy. The most common cardiomyopathy in DHF was restrictive cardiomyopathy, which is characterized by cardiac diastolic dysfunction and fibrosis. In SHF group, cardiomyopathy (57.1%) was much more prominent than other diseases. Interestingly, almost 40% of CMs were endocardial fibroelastosis (EFE). The second most common cause of SHF was arrhythmia (19%). Surprisingly, the proportion of CHDs associated with SHF was just 7.1%, a significant departure from DHF, SHF was rarely associated with simple CHDs. However, we cannot exclude the influence of few CHD case numbers in SHF group on constituent ratio of primary diseases of SHF. A small sample size of SHF case is one of the limitations in this study. Although there were just 42 SHF cases (only 3 of them were induced by CHDs), we tend to consider CM is predominant cause of SHF. Because DHF seems to occur earlier than SHF. Most of those CHDs patients were diagnosed in early stage, in this phase cardiac contractility was sufficient to maintain a relative normal LVEF and LVFS value. In that time, DHF could be found in those patients. Also, the average age of patients in SHF was significant older than that in DHF patients. But this speculation needs to be verified in future studies, especially a multi-center and larger-sample study in pediatric HF.

**TABLE 2:** Primary diseases of pediatric DHF and SHF.

Disease classification	DHF group	SHF group	Primary diseases	DHF group	SHF group
	*N* (%)	*N* (%)		*N* (%)	*N* (%)
CHDs	291 (69.1)	3 (7.1)	CCHDs	225 (53.4)	3 (7.1)
			ASD	22 (5.2)	0 (0)
			VSD	32 (7.6)	0 (0)
			PDA	12 (2.85)	0 (0)
CMs	31 (7.4)	24 (57.1)	HCM	9 (2.1)	0 (0)
			DCM	4 (1.0)	8 (19.0)
			RCM	12 (2.9)	0 (0)
			EFE	6 (1.4)	16 (38.1)
AR	25 (5.9)	8 (19.0)	AVB	7 (1.7)	3 (7.1)
			SVT	5 (1.2)	1 (2.3)
			VT	5 (1.2)	2 (4.7)
			APB	3 (0.7)	0 (0)
			Others	5 (1.2)	2 (4.7)
Multiple diseases	15 (3.6)	0 (0)	CHD, AR	11 (2.9)	0 (0)
			CHD, Myocarditis	2 (0.5)	0 (0)
			Myocarditis, AR	2 (0.5)	0 (0)
Myocarditis	9 (2.1)	1 (2.3)			
PAH	8 (1.9)	1 (2.3)			
KD	1 (0.2)	2 (4.7)			
Po-CHD	17 (4.0)	0 (0)			
Others	24 (5.7)	3 (7.1)			

*CHD, congenital heart defect; CCHD, complex congenital heart defects; ASD, atrium septal defect; VSD, ventricular septal defect; PDA, patent ductus arteriosus; CM, cardiomyopathy; HCM, hypertrophic cardiomyopathy; DCM, dilated cardiomyopathy; RCM, restrictive cardiomyopathy; EFE, endocardial fibroelastosis; AR, arrhythmia; AVB, atrioventricular block; SVT, supraventricular tachycardia; VT, Ventricular tachycardia; APB, atrial premature beat; PAH, pulmonary arterial hypertension; KD, Kawasaki disease. PO-CHD, post-operation of CHD.*

### Symptoms and Signs of Heart Failure in Children

As shown in Table 3, in 421 DHF patients, 307 (72.9%) of had tachypnea, 63.9% of those children suffered from cyanosis, and nearly 60% patients (246/421) suffered from poor appetite. Other common symptoms were feeding difficulty (decreased stamina, 42%) and diaphoresis (33.2%). Notably, hepatomegaly was found in most patients (87.4%). Similar with DHF, poor appetite (57.1%), tachypnea (54.7%), diaphoresis (45.2%), and hepatomegaly (85.7%) were the most common symptoms and signs in children with SHF. In compared with SHF, cyanosis (63.9 vs. 35.7%), feeding difficulty (decreased stamina, 42 vs. 16.7%) and be fidgety (33.3 vs. 11.9%) were more specific in DHF, *p* < 0.01. On the contrary, pale complexion (15.9 vs. 35.7%), fatigue (12.3 vs. 33.3%), and edema (8.1 vs. 23.8%) were more common symptoms in SHF, when compared to DHF patients, *p* < 0.01. Early diagnosis and effective treatment of HF remain significant challenges. Also, diastolic HF is an important but as yet poorly elucidated topic in the pediatric literature. Data from our study indicated that in children with heart diseases, pediatricians should be alert of DHF when symptoms such as tachypnea, cyanosis and poor appetite, and sign like hepatomegaly are found in their patients.

**TABLE 3:** Symptoms and signs of DHF and SHF in children.

Symptoms and signs	DHF group	SHF group	χ^2^	*p*-Value
	*N* (%)	*N* (%)		
Total cases	421 (100)	42 (100)		
Listlessness	235 (55.8)	23 (54.8)	0.02	0.90
Poor appetite	246 (58.4)	24 (57.1)	0.03	0.87
Pale complexion	67 (15.9)	15 (35.7)	10.27	0.00
Be fidgety	140 (33.3)	5 (11.9)	8.10	0.00
Edema	34 (8.1)	10 (23.8)	10.99	0.00
Tachypnea	307 (72.9)	23 (54.7)	6.15	0.01
Fatigue	52 (12.3)	14 (33.3)	13.76	0.00
Diaphoresis	140 (33.2)	19 (45.2)	2.43	0.12
Oliguria	77 (18.2)	12 (28.6)	2.60	0.11
Feeding difficulty	177 (42.0)	7 (16.7)	10.27	0.00
Chest tightness	23 (5.5)	3 (7.1)	0.20	0.65
Palpitation	24 (5.7)	9 (21.4)	13.71	0.00
Vomit	18 (4.2)	8 (19.0)	15.72	0.00
Cough	302 (71.7)	26 (61.9)	1.78	0.18
Fever	118 (28.0)	11 (26.2)	0.06	0.80
Cyanosis	269 (63.9)	15 (35.7)	12.80	0.00
Hepatomegaly	368 (87.4)	36 (85.7)	0.10	0.75
Jugular vein distension	8 (1.9)	1 (2.3)	0.05	0.83

*DHF, diastolic heart failure; SHF, systolic heart failure.*

### B-Type Natriuretic Peptide Detection in 1,046 Hospitalized Patients

**FIGURE 1:**
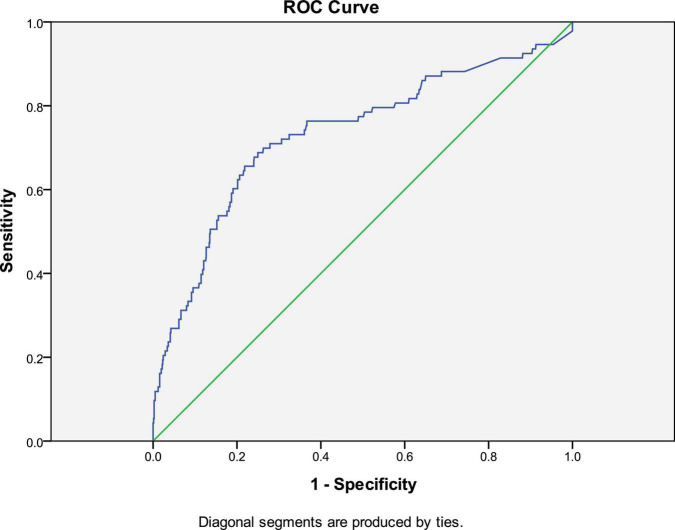
ROC curve in diagnosis of children DHF with BNP cut-off value as 100 pg/ml.

In adult HF, the most common biomarker is BNP and/or its precursor protein N-terminal prohormone of BNP (NT-proBNP) (12). BNP is used for diagnosis, treatment monitoring, and prognosis (13). Their value varies according to age group and etiology of HF. However, cut-off value of BNP in pediatric HF has not been well studied. In our study, the mean value of BNP in 130 DHF patients was 97.83 ± 217.97 pg/ml. The cut-off value in adult (lower than 75 years) is 100 pg/ml. The sensitivity of BNP greater than 100 pg/ml in diagnosis of DHF was just 0.37, the specificity of it was 0.86, as shown in Tables 4, 5. Area under ROC curve (AUC) of BNP in diagnosis of DHF was 0.733, as indicated by Figure 1. Meanwhile, normal range of BNP in newborns are still poorly understood, it is commonly higher than that in elder children. Mammalian hearts undergo maturation period after birth. This process is characterized by structural, gene expression, metabolic, and functional specializations in cardiomyocytes as the heart transits from fetal to adult states. Therefore, it is more complex to set up cut off value of BNP in children HF.

**TABLE 4:** Plasma BNP levels in DHF and SHF patients.

	DHF group	SHF group	χ^2^	*p*-Value
Case numbers	130	20	/	/
A/N	48/82	12/8	3.85	0.50
Mean value (pg/ml)	93.95 ± 208.82	110.25 ± 183.34	0.330	0.74

*A, abnormal cases; N, normal cases; DHF, diastolic heart failure; SHF, systolic heart failure; BNP, B-type natriuretic peptide.*

**TABLE 5:** Sensitivity and specificity of BNP cut-off value at 100 pg/ml in diagnosis of pediatric DHF and SHF.

Heart function	Normal BNP (*n*)	Abnormal BNP (*n*)	Sensitivity	Specificity
Normal	765	64	/	/
DHF	82	48	0.37	0.86
SHF	8	12	0.6	0.84

*n means case numbers. DHF, diastolic heart failure; SHF, systolic heart failure; BNP, B-type natriuretic peptide.*

### Treatment Effect of Diastolic Heart Failure in Children

The treatment of heart failure in children depends on the underlying cause and the child’s age (14). Therefore, in this section, heart function was evaluated by echocardiography in DHF patients 3 months after the discharge. A total number of 101 patients reexamined cardiac echo, most of those patients showed partially recovery of diastolic heart function, as indicated by early wave/late wave (E/A) ratio, isovolumic relaxation time (IVRT), and left ventricular diastolic dysfunction (LVDD) (Table 6). Among those 101 patients, abnormal E/A ratio, IVRT, and LVDD could be found that more than 50% of those DHF children’s diastolic function indexes [E/A ratio (30/101 to 85/101) and IVRT (30/101 to 84/101)] partially recovered. An increase of 30% of those 101 patients improved in LVDD after treatment (20/101 to 55/101). In those 101 patients, 59 children underwent medical therapy and surgery or interventional therapy, the rest of patients received medical therapy only. As shown in Table 7, in operation group, the proportion of normal E/A value increased from 18.5% (11/59, before treatment) to 93.2% (55/59, post-treatment), with a jump more than 70%, and it was significantly higher than that of non-operation group, with an increase of 26% (from 45.2%, 19/42 to 71.4%, 30/42). Similar results were seen in measurement of IVRT (57.6% leap in operation group vs. 47.6% in non-operation group). An increase tendency was found in LVDD, but there was no statistical difference between operation and non-operation group. Meanwhile, treatment effect was also evaluated between CMs caused DHF group (8 patients) and non-CMs caused DHF group (93 patients). As illustrated in Table 7, diastolic function indicators, such as E/A ratio (from 27.9 to 87.1%), IVRT (from 20.1 to 87.1%) and LVDD (from 19.4 to 55.9%) improved markedly in non-CMs DHF patients after therapies. In non-CMs induced DHF children, cardiac diastolic function did not significantly improve with medical treatment. Medications for those HF patients included ACEI (angiotensin-converting enzyme inhibitor), β blocker, digitalis, calcium channel blocker, dopamine, and diuretic. Data of our study indicated that the mortality might differ in DHF and SHF. In the present study, our data showed that most of DHF patients’ heart function recovered after treatment, especially in those CHDs induced DHF patients.

**TABLE 6:** Case numbers of diastolic function indicators in DHF patients, before and after treatment.

Indicator of DD	Pre-treatment		Post-treatment			
	Normal	Abnormal	Normal	Abnormal	χ^2^	*p*-Value
E/A	30	71	85	16	62.2	0.00
IVRT	30	71	84	17	59.9	0.00
LVDD	20	81	55	46	26.65	0.00

*DD, diastolic dysfunction; E, peak E wave velocity; A, peak E wave velocity; IVRT, Isovolumic relaxation time; LVDD, left ventricle diastolic diameter.*

**TABLE 7:** Cardiac diastolic function evaluation of DHF patients before and after therapies.

Group	E/A normal case numbers		IVRT normal case numbers		LVDD normal case numbers	
	Before	After	Before	After	Before	After
Operation group (*n* = 59)	11	55	21	55	10	37
Non-operation group (*n* = 42)	19	30	9	29	10	18
χ^2^	17.12		8.082		7.148	
*p*-Value	0.00		0.004		0.008	
CMs group (*n* = 8)	4	4	2	3	2	3
Non-CMs group (*n* = 93)	26	81	28	81	18	52
χ^2^	/		/		/	
*p*-Value	0.002		0.002		0.228	

*In comparison between CMs induced DHF group and non-CMs induced DHF, as n < 5, here we used Fish’s Exact Test. E, peak E wave velocity; A, peak E wave velocity; IVRT, Isovolumic relaxation time; LVDD, left ventricle diastolic diameter.*

## Discussion

Approximately half the adult patients with newly diagnosed heart failure have “diastolic heart failure” with preserved systolic function (15). The prevalence of diastolic heart failure in pediatric patients with congenital and acquired heart diseases is unknown (16). In addition, the clinical features of DHF have been rarely reported. Our study summarized the clinical manifestation of 421 pediatric DHF patients, from general information, causes, symptoms and signs, BNP level to treatment effects. However, there are also several shortcomings in our study, such as the clinical data were obtained before 2015, a small number of SHF cases were collected. This is mainly because our medical record system got updated in 2015, and a much smaller number of DHF patients could be collected since 2015.

We found the average age of DHF children was younger than that of SHF patients. This could be explained by the age differences among those primary cardiovascular diseases. As the use of three- or four-dimension ultrasound in prenatal examination and improved screening for CHDs postnatally, CHDs are now more frequently to be found at an earlier age. Alternatively, early diagnosis of CMs is always difficult to accomplish (17). Notably, EFE dominated SHF primary diseases, accounting for 38.1% of CMs, and most of them were infants. EFE is usually difficult to identify with dilated cardiomyopathy (DCM), especially in infants (18). Cardiac MRI and genetic analysis are helpful in differential diagnosis of EFE and DCM (19, 20), however, cMRI and gene sequencing were not generally available before 2015. Therefore, in our cases, those EFE might be confused with idiopathic DCM (20). Consistent with prior publications, our data revealed that CHDs and CMs were still the most common causes of pediatric heart failure (1). In addition, CHDs were dominant causes of DHF, and in SHF, most of those children were suffered from CMs.

Our data showed that tachypnea and hepatomegaly were the most common symptom and sign in pediatric heart failure, which are considered as the most reliable indicators in diagnosis and degree assessment (The ROSS Score) (11) of pediatric HF. Decrease of effective circulating blood volume is the main pathological disorder in HF. Most of HF symptoms are associated with insufficient blood perfusion, such as pale, feeding difficulty, sweating, etc. (21). In this study, we found that symptoms like cyanosis, feeding difficulty and be fidgety were more specific in pediatric DHF, and pale complexion, fatigue, and edema were more common in SHF patients. However, these finding are very subjective as they are made by the physician and not easily defined.

B-type natriuretic peptide has been considered a primary and one of the most valuable markers in adult heart failure in many aspects, like diagnosis, degree assessment, prognosis, and treatment efficiency evaluation (22, 23). But, in pediatric heart failure, the role of BNP still needs to be determined (24). In healthy infants and children, levels of BNP are elevated in the first few days after birth. Thereafter, its levels decline and remain relatively constant throughout childhood (25). There is no unified cut-off standard of BNP in pediatric heart failure. Our data suggested that level of 100 pg/ml of BNP was insensitive in use of pediatric HF evaluation. Scoot et al. reported that in children with moderately symptomatic HF, BNP ≥ 140 pg/mL and age >2 years identified subjects at higher risk for worse outcome. Further validation is needed to determine the BNP levels necessary to stratify risk in other pediatric cohorts.

At present, prognosis of DHF in adults is poor and irreversible, but in pediatric patients, treatment effect of DHF seems more promising, mainly because of two reasons: (1) most causes of pediatric DHF were congenital heart defects, which is more curable in compared with cardiomyopathy and ischemic heart diseases; (2) most children with DHF were at younger age, those unmatured hearts and their functions were more recoverable.

Pediatric heart failure is still largely unknown. A major future direction to improve treatment of heart failure in children is to obtain sufficient data from well-designed trials with adequate power to support treatment recommendations.

## Limitations

There are some limitations of the present study. (1) A small sample size of SHF case, which means we cannot exclude the influence of few CHD case numbers in SHF group on constituent ratio of primary diseases of SHF. (2) The importance of genetic tests in CM patients has been increasingly recognized in recent years. Another limitation of this present study is that none of our CM patients completed genetic testing. The main reason was that most of the parents could not afford the genetic test, the test fee was quite expensive a decades ago. Fortunately, with the development of the economy and the cut of test price, more and more CM children underwent genetic test in our center, also in whole China. In future studies, we hope to report role of genetic factors in pediatric CMs and DHF. (3) Meanwhile, it is a pity that we failed to follow-up all our patients 3 months after discharge, in those 101 patients reexamined cardiac echo, none of them died in the first 3 months. That means we fail to compare the treatment between those patients who died and those who did not. We are expecting to compare that part in our prospective study in the near future.

## Data Availability Statement

The original contributions presented in the study are included in the article/Supplementary Material, further inquiries can be directed to the corresponding authors.

## Ethics Statement

The studies involving human participants were reviewed and approved by the Institutional Review Board of Children’s Hospital of Chongqing Medical University. Written informed consent for participation was not required for this study in accordance with the national legislation and the institutional requirements.

## Author Contributions

WX, TL, and JT contributed to the conception and design of this study. BP and WX organized the data and performed the statistical analysis. BP and DH drafted the manuscript. BP, DH, and HS wrote sections of the manuscript. All authors contributed to manuscript revision, read, and approved the submitted version.

## Conflict of Interest

The authors declare that the research was conducted in the absence of any commercial or financial relationships that could be construed as a potential conflict of interest.

## Publisher’s Note

All claims expressed in this article are solely those of the authors and do not necessarily represent those of their affiliated organizations, or those of the publisher, the editors and the reviewers. Any product that may be evaluated in this article, or claim that may be made by its manufacturer, is not guaranteed or endorsed by the publisher.
