# Supplementary material for: Pediatric Diastolic Heart Failure: Clinical Features Description of 421 Cases
Source: Front Pediatr. 2022 May 2;10:846408. doi: 10.3389/fped.2022.846408 (PMC9108191; doi:10.3389/fped.2022.846408)
Supplement: Supplementary file 1 [file Table_1.pdf]

Table S1 Information of 225 Complex CHDs cases

Case No.9 - 120	Type of CCHD	Case No. 121- 233	Type of CCHD
	ASD, PDA, VSD		TOF
	ASD, VSD		TOF
	ASD, ASD		ASD, VSD, MGA
	PDA, ASD, MS		TOF
	PDA, MI		TA, PS, RVD, VSD, ASD, PDA
	PDA, MS		DORV, ASD
	PDA, AS		DORV, VSD, PDA
	PDA, AS		DORV, ASD, VSD, PDA
	PDA, AS		DORV, VSD
	TOF, PDA		DORV, ASD, VSD, PDA, PS
	TOF, PDA, ASD		DORV, ASD, VSD, PDA
	TOF, ASD, RAA		DORV, ASD, VSD, PDA, PS
	VAD, PDA		DORV, AS, VSD
	VSD, ASD, PDA		DORV, MGA, ASD, VSD, PDA
	VSD, ASD, AS		DORV, ASD, VSD, PDA, PS
	VSD, PDA, MI		DORV, VSD
	VSD, PDA, PS		DORV, VSD, PDA, MGA
	VSD, PDA, AS, PLSVC		DORV, VSD, PDA, PS
	VSD, PDA, AS, MS		DORV, ASD, VSD, PDA, SVAs
	VSD, MS, PDA		DORV, CAVSD, PS, MGA, PDA
	VSD, TI, MS		DORV, RVD, ASD, VSD
	VSD, AS		ASD, PDA, RVD
	VSD, AS		RVD, PVA, TS, ASD, PDA
	VSD, AS		RVD, AS
	VSD, AS, PDA		RVD, TS, PVA, ASD, PDA
	VSD, AS		RVD, ASD
	APVC, ASD		ASD, VSD
	APVC, ASD		TOF
	APVC, ASD, CT		ASD, VSD
	ASD, VSD, PDA		TOF
	SA, CAVSD (TYPE A), TGA, PS, PDA		CoA, VSD
	MI, PDA		TOF
	MI, ASD		PDA, CoA
	MS, AS, PDA		VSD, PDA, CoA
	TOF		VSD, PDA
	TOF, ASD		VSD, PDA
	TOF, ASD		TOF
	TOF, ASD		TOF
	TOF, ASD, ALPA		DORV, CoA, ASD
	TOF, PDA, ASD		ASD, PDA
	TOF		SVAs, PDA
	ASD, PDA		CoA, VSD
	PVA, PDA, ASD		CoA, PDA
	PVA, VSD, PDA, ASD		LVD, MVD, IAA, ASD, PDA, PS
	PVA, VSD, PDA		HLHS, ASD
	PA, RVD, ASD, PDA		DOLV, ASD, VSD, PDA, PS

PS, ASD	TOF
PS, ASD	HLHS, ASD
PS, ASD	HLHS, MI
PS, ASD	ASD, VSD
PS, ASD	ASD, VSD
PS, PDA	ASD, VSD
PS, ASD	ASD, PDA
PVA, ASD, VSD, PDA	ASD, PDA
PVA, VSD, ASD, PDA, LVD	ASD, PDA
PVA, VSD, RAA	ASD, PDA
PVA, VSD, PDA, ASD, ASa	ASD, PDA
PVA, RVD, PDA, ASD, VSD	ASD, PDA
PS, MI	ASD, PDA
PS, ASD, LVD	ASD, VSD
PS, ASD, PDA	ASD, VSD
ASD, LVDC	ASD, VSD
ASD, MS	ASD, VSD
ASD, PS	ASD, VSD
ASD, VSD	ASD, VSD
VSD, PDA	ASD, PDA
PS, ASD, VSD, PDA	ASD, VSD, PDA
APVC, VSD, ASD, PDA	ASD, PDA
TOF	PDA, CoA
TOF	ASD, VSD, PDA
ASD, VSD	VSD, PDA
DORV, VSD, PDA, PS	ASD, VSD, PDA, PLSVC
DORV, VSD, ASD, AS	ASD, VSD
AS, PDA	ASD, VSD
TOF	ASD, VSD
TOF	ASD, VSD
TOF	ASD, VSD
DORV, CAVSD, PS	ASD, VSD
PS, ASD	ASD, VSD
VSD, PDA, PVA, RAA, MGA, Dextrocardia	ASD, VSD
Dextrocardia, VSD	ASD, VSD, PDA
DCRV, VSD	ASD, VSD, PDA
ASD, VSD	ASD, VSD, PDA
TA, ASD, VSD	ASD, VSD
TA, ASD, VSD, PS	ASD, VSD
TGA, ASD, PDA	ASD, VSD
TGA, ASD	ASD, VSD
CAVSD	VSD, PDA
CVASD, PS	VSD, PDA
CVASD	VSD, PS
TAPVC, ASD	ASD, VSD
TAPVC, ASD	ASD, VSD, PDA
TAPVC, ASD	ASD, VSD
TGA, ASD, PDA, PS	ASD, VSD
TGA, ASD, VSD, PS	ASD, VSD
TGA, VSD, PDA	ASD, VSD

TGA, VSD, PS	ASD, VSD
CAVSD, SA, PS	ASD, VSD, PDA
CAVSD, SA	VSD, PDA
TAPVC, ASD, LVD	VSD, PDA
TAPVC, ASD, LVD, PDA	ASD, PDA
TAPVC, ASD, PDA	ASD, VSD
TAPVC, ASD	ASD, VSD, PDA
TAPVC, ASD	ASD, VSD
TAPVC, ASD	ASD, VSD, PDA
TAPVC, ASD	ASD, PDA
TAPVC, ASD, PDA	ASD, VSD, PDA
TAPVC, VSD	VSD, PDA
TAPVC, ASD, LVD, PDA	ASD, VSD, PDA
TAPVC, ASD	ASD, VSD, PDA
TAPVC, ASD	ASD, VSD, PDA, PS
DORV, VSD, PS	VSD, PDA
	ASD, TAPVC

A total number of 225 CCHD cases were collected, and numbered from 9 to 233. Abbreviation of CCHDs are as followed:

ALPA, absence of left pulmonary artery; APVC, anomalous pulmonary venous connection; AS, aortic stenosis; ASa, aortic saddle; ASD, atrium septal defect; CAVSD, complete atrioventricular septal defect; CoA, coarctation of aorta; CT: cor triatriatum; DCRV, double-chambered right ventricle; DORV, double outlet of right ventricle; DOLV, double outlet of left ventricle; HLHS, hypoplastic left-heart syndrome; IAA, interrupted aortic arch; LVD, left ventricular dysplasia; LVDC, left ventricle double-chambered; MI, mitral insufficiency; MS, mitral stenosis; MGA, malposition of the great arteries; MVD, mitral valve dysplasia; PDA, patent ductus arteriosus; PLSVC, persistent left superior vena cava; PS, pulmonary stenosis; PVA, pulmonary valve atresia; RAA, right aortic arch; RVD, right ventricular dysplasia; SA, single atrium; SVAs, supravalvular aortic stenosis; TA, tricuspid atresia; TAPVC, total anomalous pulmonary venous connection; TGA, transposition of great arteries; TOF, tetralogy of Fallot; TS, tricuspid stenosis; VSD, ventricular septal defect.
